# Uranium Biominerals Precipitated by an Environmental Isolate of *Serratia* under Anaerobic Conditions

**DOI:** 10.1371/journal.pone.0132392

**Published:** 2015-07-01

**Authors:** Laura Newsome, Katherine Morris, Jonathan. R. Lloyd

**Affiliations:** Research Centre for Radwaste Disposal and Williamson Research Centre, School of Earth, Atmospheric and Environmental Sciences, University of Manchester, Oxford Road, Manchester, United Kingdom; Belgian Nuclear Research Centre SCK•CEN, BELGIUM

## Abstract

Stimulating the microbially-mediated precipitation of uranium biominerals may be used to treat groundwater contamination at nuclear sites. The majority of studies to date have focussed on the reductive precipitation of uranium as U(IV) by U(VI)- and Fe(III)-reducing bacteria such as *Geobacter* and *Shewanella* species, although other mechanisms of uranium removal from solution can occur, including the precipitation of uranyl phosphates via bacterial phosphatase activity. Here we present the results of uranium biomineralisation experiments using an isolate of *Serratia* obtained from a sediment sample representative of the Sellafield nuclear site, UK. When supplied with glycerol phosphate, this *Serratia* strain was able to precipitate 1 mM of soluble U(VI) as uranyl phosphate minerals from the autunite group, under anaerobic and fermentative conditions. Under phosphate-limited anaerobic conditions and with glycerol as the electron donor, non-growing *Serratia* cells could precipitate 0.5 mM of uranium supplied as soluble U(VI), via reduction to nano-crystalline U(IV) uraninite. Some evidence for the reduction of solid phase uranyl(VI) phosphate was also observed. This study highlights the potential for *Serratia* and related species to play a role in the bioremediation of uranium contamination, via a range of different metabolic pathways, dependent on culturing or *in situ* conditions.

## Introduction

*Serratia* species are Gram-negative facultative anaerobes; they are ubiquitous and can be found in many natural environments [1]. In terms of bioremediation of subsurface groundwater contamination, organisms of this genus are of interest due to their ability to precipitate uranyl phosphate biominerals. This feature was first discovered in the early 1990s [2,3] using laboratory cultures of *Citrobacter* (since reclassified as a *Serratia* species [4]) under aerobic conditions in a medium supplemented with glycerol phosphate. Later investigations showed that *Serratia* species can over-produce phosphatase enzymes, which cleave the glycerol phosphate molecule causing the release of inorganic phosphate to solution and consequently the precipitation of uranyl(VI) phosphate biominerals [5]. Although most work on *in situ* microbial uranium(VI) remediation has focussed on reductive precipitation of U(IV) by Fe(III)-reducing bacteria [6] such as *Geobacter* and *Shewanella* species [7] or sulfate-reducing bacteria [8], the potential for uranium-phosphate biomineralisation by *Serratia* species offers some distinct advantages. In particular, the mineral end product uranyl phosphate is not susceptible to oxidative remobilisation, unlike the products of microbial U(VI) reduction which can be reoxidised to mobile U(VI) by oxygen or nitrate e.g. [9–11]. Stimulating microbial uranium phosphate mineral precipitation via glycerol phosphate addition *in situ* would be advantageous over simply adding inorganic phosphate to the subsurface, as it avoids problems caused by clogging of the injection location due to the rapid precipitation of metal phosphate phases [11,12].

As well as growing via aerobic respiration, *Serratia* species have been shown to respire nitrate and Fe(III)_aq_ anaerobically, coupled to the use of a number of different electron donors (acetate, lactate, formate, ethanol, glucose, glycerol), and to function from pH 3 to pH 9 [13,14]. *Serratia* species are also known to ferment various organic compounds and utilise them as their sole carbon source, including glucose and glycerol, and most strains can grow readily at 4–5°C [1]. These characteristics suggest that *Serratia* species may be capable of mediating many biogeochemical processes in varied subsurface conditions, and could therefore play an important role in stimulated U(VI) bioremediation. Supporting evidence for this can be found from laboratory and field studies. Bacteria closely related to *Serratia marcescens* have been isolated from sandstones containing 0.1% U_3_O_8_, and were observed to tolerate millimolar concentrations of U(VI) [15]. A nitrogen fixing gene, *nifH*, from *Serratia marcescens* was also detected in groundwater sampled from a well stimulated by ethanol during a bioremediation trial at the US Department of Energy (DOE) Oak Ridge site [16], suggesting that it was functioning during biostimulated U(VI) reduction. Furthermore, an investigation into the radiation tolerance of a U(VI)-phosphate precipitating *Serratia* species showed that it was able to produce phosphatases at a gamma dose of over 1000 Gy [17].

A *Serratia* species closely related to *Serratia liquefaciens* (99% 16S rRNA gene sequence homology) was isolated previously from sediments representative of the Sellafield nuclear site, Cumbria, UK, [14]. This *Serratia* species offered an interesting opportunity to compare whether an environmental isolate representative of this genus and present in sediments representative of a nuclear site, can biomineralise uranyl phosphates and/or reduce U(VI). Here we investigated this in a series of pure culture experiments, under anaerobic conditions using both growing and resting cells. In brief, this *Serratia* strain was readily able to precipitate uranyl phosphates under anaerobic conditions; in addition evidence for U(VI) reduction was observed in some experiments, but this occurred at a slower rate and to a lesser extent than uranyl phosphate mineral precipitation. The ability to fine tune uranium bioremediation end points has implications for the long-term stewardship of land contaminated by radioactive waste.

## Materials and Methods

### Cultivation and growth of a *Serratia* species

A *Serratia* species, previously isolated from Sellafield sediments [14] and maintained in our laboratory culture collection, was inoculated into 50 mL LB broth medium (Sigma) and incubated at 30°C on an orbital shaker for 24 hours. One millilitre of the LB medium culture was inoculated into 100 mL of sterile freshwater minimal medium [7,14] at pH 7, driven anaerobic by flushing with an N_2_ and CO_2_ gas mix (80:20), and containing 10 mM glycerol as the electron donor and 20 mM fumarate as the electron acceptor. This medium contained 30 mM bicarbonate and 4.3 mM phosphate. Cultures were incubated in sealed bottles in the dark at 30°C. Controls contained no added electron acceptor. Growth was monitored by measuring the optical density at 600 nm. The stock was maintained by adding 1 mL of the culture via a sterile syringe degassed with N_2_ to 100 mL of the anaerobic sterile freshwater minimal medium.

### Uranium solubility in freshwater minimal medium

U(VI) at 0.5 mM or 1.0 mM was added to 10 mL anaerobic freshwater minimal medium from a uranyl chloride stock solution. The concentration of U(VI) in solution was measured over two to five days using the bromo-PADAP colorimetric assay [18]. This colorimetric assay routinely measures U(VI) concentrations from 0 to 125 μM in a standard 1 cm path length cell, therefore samples were diluted to within this range before analysis. Under these conditions the solution chemistry contained no interfering reagents at the point of analysis. These experiments were repeated with sodium phosphate excluded from the medium and with glycerol phosphate or glycerol included as appropriate. Additional uranium solubility experiments were conducted with the 4.3 mM sodium phosphate in the freshwater minimal medium replaced by 1.4 mM sodium trimetaphosphate (Na_3_P_3_O_9_, Sigma) to minimise abiotic uranium-phosphate mineral precipitation [19].

### Uranium-phosphate biomineralisation

The *Serratia* species was maintained in anaerobic freshwater minimal medium with 10 mM glycerol as the electron donor and 20 mM fumarate as the electron acceptor. Stationary phase cells were inoculated at 1% (vol/vol) into 100 mL anaerobic freshwater minimal medium containing 10 mM glycerol phosphate, 20 mM fumarate and 1 mM U(VI), excluding sodium phosphate to avoid abiotic uranium-phosphate mineral precipitation. Experiments were conducted in triplicate. A control incubation contained no added electron acceptor (fumarate). Growth was monitored by measuring the optical density at 600 nm. Concentrations of aqueous U(VI) were measured via bromo-PADAP and phosphate was monitored using ion chromatography (Dionex ICS 5000).

### Microbial uranium(VI) reduction

Growing cell cultures – 1 mL of a stationary phase *Serratia* species culture was inoculated into 100 mL anaerobic freshwater minimal medium containing 10 mM glycerol, 20 mM fumarate and 1 mM U(VI), with the sodium phosphate replaced with sodium trimetaphosphate to avoid abiotic uranium-phosphate mineral precipitation. A control contained no added fumarate. Growth was monitored by measuring the optical density at 600 nm and concentrations of aqueous U(VI) were measured using bromo-PADAP.

Resting cell suspensions–Cells of the *Serratia* species were grown in anaerobic freshwater minimal medium with 10 mM glycerol and 20 mM fumarate. The cells were harvested at the late-logarithmic phase via centrifugation, washed twice in anaerobic 30 mM bicarbonate buffer at pH 7, then suspended in 10 mL bicarbonate buffer to an optical density at 600 nm of around 1.0. Uranium(VI) at 0.5 mM was added as the sole electron acceptor. Electron donors included: 10 mM glycerol; 10 mM glycerol plus 0.1 mM AQDS (a humic analogue and electron shuttle); 10 mM glycerol and 20 mM fumarate (an additional electron acceptor); or with approximately 40 mL of H_2_ in the headspace. The control cultures contained no added electron donor. U(VI) in solution was monitored using the bromo-PADAP assay.

Solid phase U(VI) reduction–approximately 5 mg of the precipitate from the uranium biomineralisation experiment (autunite) was separated from the growth medium by centrifugation, washed twice with anaerobic deionised water, then suspended in a 30 mM bicarbonate buffer at pH 7 containing 10 mM glycerol as the electron donor. Resting cell suspensions of the *Serratia* species were prepared as above, and were added to achieve a biomass optical density at 600 nm of around 1.0. The solid U(VI) bearing mineral was the sole electron acceptor included in this system.

### Mineral identification

The composition of the mineral precipitates was determined using X-ray diffraction (XRD) (Bruker D8 Advance) and transmission electron microscopy (TEM) with selected area electron diffraction (SAED) (Philips CM200 FEG TEM equipped with a field emission gun). Information on the oxidation state and co-ordination environment of uranium was obtained via X-ray absorption spectroscopy (XAS) at the Diamond Light Source, Harwell. Samples were prepared for XAS by dilution with cellulose powder, then formation into pellets which were stored at -80°C under anaerobic conditions before analysis. Uranium L_III_-edge spectra were collected in transmission mode with an yttrium foil used as an in-line reference standard. Data were calibrated, background subtracted and normalised using ATHENA [20]. Spectra were compared to standards comprising U(IV) as uraninite and U(VI) as a uranyl carbonate complex obtained from the Actinide Reference Database for Spectroscopy [21–23]. ARTEMIS [20] was used to fit extended X-ray absorption fine structure (EXAFS) spectra. Fits were obtained independently by shell by shell fitting, with additional shells only included if they made a statistically significant change to the model [24].

## Results and Discussion

### Growth of a *Serratia* species isolated from Sellafield sediments

The *Serratia* species was able to grow under anaerobic conditions with glycerol or glycerol phosphate as an electron donor and fumarate as the electron acceptor, reaching the late logarithmic phase of growth after approximately 55 hours (S1 Fig). No growth was observed in the controls without fumarate after seven days.

### Uranium solubility

U(VI) at 0.5 mM and 1.0 mM precipitated rapidly from solution in the freshwater minimal medium forming a yellow precipitate, presumably due to abiotic uranium phosphate precipitation [12]. Solubility experiments were then conducted in the freshwater minimal medium with the sodium phosphate excluded and 1 mM U(VI) remained in solution, including when 10 mM glycerol phosphate or 10 mM glycerol were included in the medium. These results were supported by PHREEQC calculations [25] that confirmed U(VI) was soluble under these conditions. Further solubility experiments were conducted replacing the sodium phosphate in the freshwater minimal medium with sodium trimetaphosphate. Use of long-chain polyphosphates such as sodium trimetaphosphate has been shown to prevent abiotic uranium phosphate precipitation as they release orthophosphate slowly via step-wise abiotic or enzymatic hydrolysis [26–28]. Results confirmed that U(VI) at 1 mM remained in solution in the freshwater minimal medium containing sodium trimetaphosphate.

### Uranium-phosphate biomineralisation

**Fig 1:**
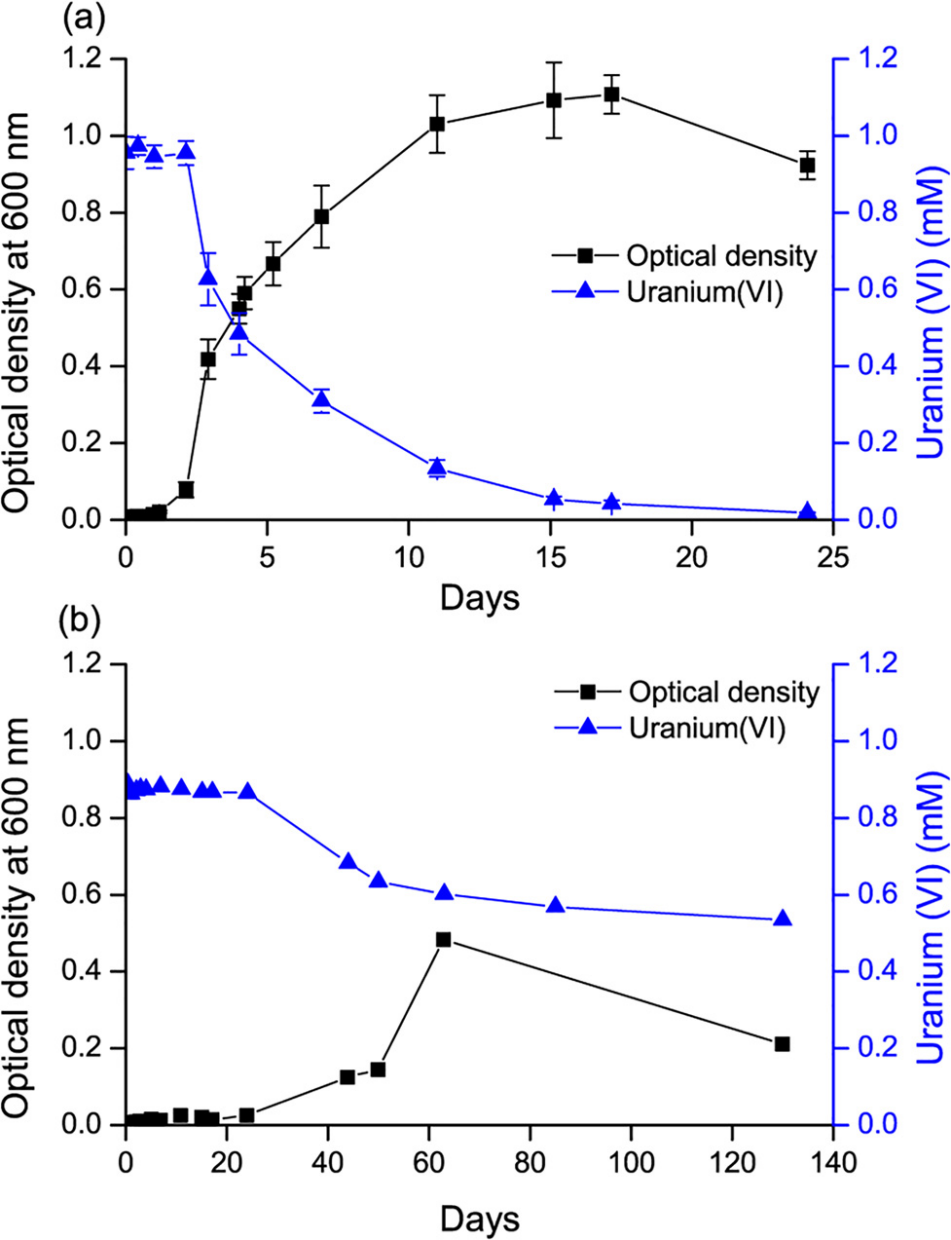
Monitoring of uranium-phosphate biomineralisation with *Serratia*. (a) With glycerol phosphate as the electron donor and fumarate as the electron acceptor, increasing optical density and U mineral precipitation. Each point represents the average of three replicates with error bars ± 1 standard deviation. (b) With glycerol phosphate and U(VI) as the sole electron acceptor and with comparatively delayed onset of cell growth and U precipitation. This control sample was a single measurement.

**Fig 2:**
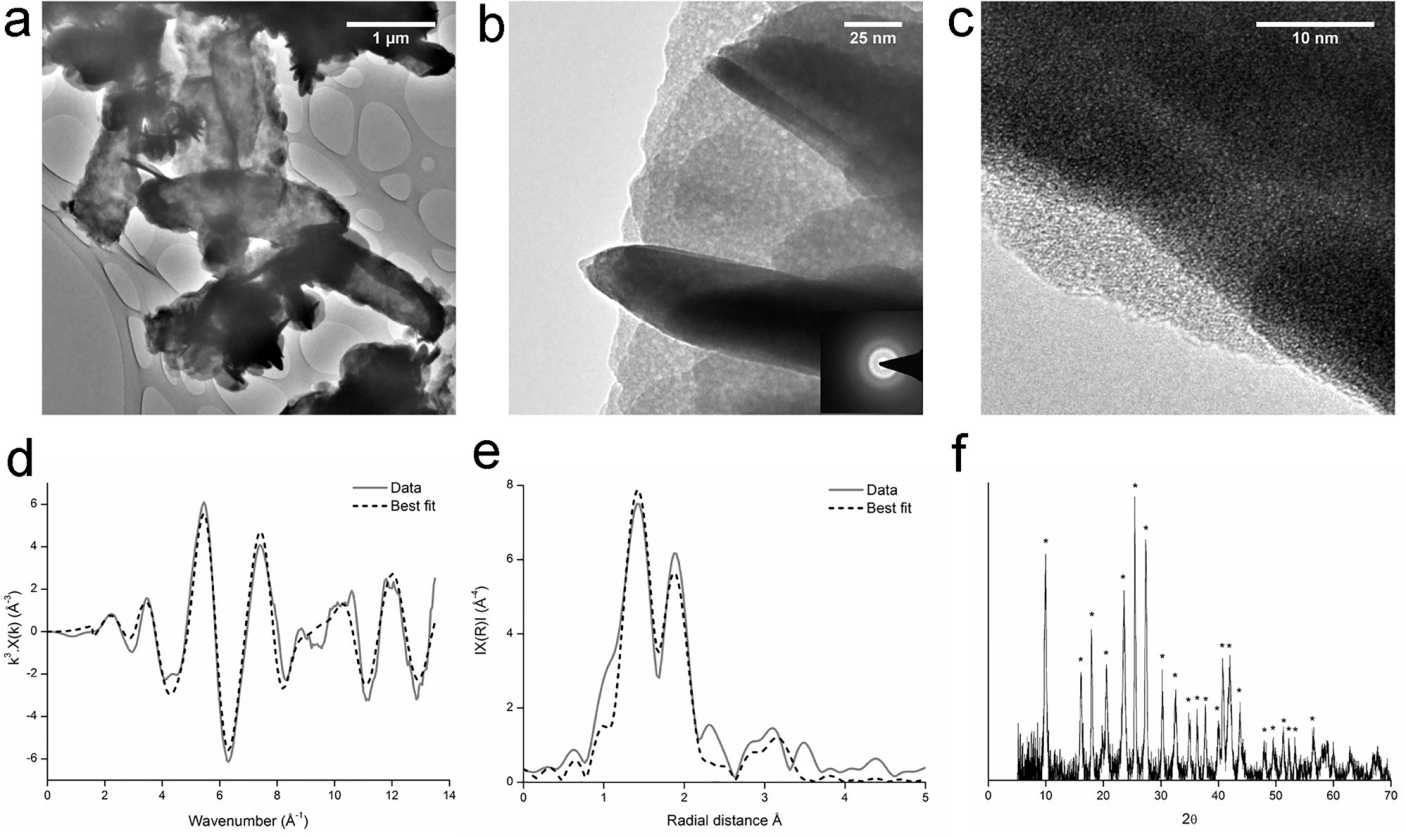
Uranium-phosphate biomineralisation experiment: TEM images (a, b, c), k^3^ weighted EXAFS data (d), non-phase shift corrected Fourier transform of EXAFS data (e) and XRD spectra (f). Dashed lines in XAS spectra represent the best fit of the data. * are peaks from uranyl phosphates (S6 Fig shows the peak pattern). Experiments were conducted in an anaerobic freshwater minimal medium with glycerol phosphate as the electron donor and both fumarate and U(VI) as electron acceptors. Results confirmed the precipitate to be a uranyl phosphate biomineral of the autunite group.

The *Serratia* species was incubated in anaerobic freshwater minimal medium with 10 mM glycerol phosphate, 1 mM U(VI) and 20 mM fumarate, to assess whether it could facilitate the precipitation of uranyl phosphate biominerals under these conditions. After a lag phase of approximately 50 hours, the optical density began to increase, and U(VI) was removed from solution (Fig 1A). The rates of U(VI) removal observed were broadly comparable with other studies [29,30], although differences in biogeochemical conditions preclude a direct comparison. Phosphate was released to solution concurrently (S2 Fig). A cream coloured precipitate formed which was identified as containing autunite group uranyl phosphates (Fig 2). TEM images showed cells coated in dense agglomerations of sheets of mineral, sometimes folded up into rolls (Fig 2). The lack of clearly defined peaks in the SAED spectrum suggested that the mineral phases were amorphous, and this was supported by high resolution images showing the mineral to be unstructured at the nanoscale. The discrepancy between the well-defined XRD peaks and the amorphous SAED pattern may be due to the autunite rapidly dehydrating in air to meta-autunite [31] during the TEM analysis, despite the maintenance of anaerobic conditions during sample drying and transport.

Results for the control cultures which contained U(VI) as the sole electron acceptor and glycerol phosphate as an electron donor showed that initially no growth was observed, and U(VI) concentrations remained constant (Fig 1B). Continued monitoring of this “no fumarate” control revealed that the optical density at 600 nm began to increase after approximately 24 days, and U(VI) concentrations started to decrease. After 130 days, approximately 50% of the added U(VI) had been removed from solution, and a cream coloured precipitate was observed, although U(VI) removal rates had decreased. Given that U(VI) was the sole electron acceptor in this system, we hypothesised that the mineral precipitate might contain U(IV). However, XRD results found the mineral to be a uranyl(VI) phosphate from the autunite group (S3 Fig). TEM images and SAED patterns were similar to those from the uranium-phosphate biomineralisation experiments (S3 Fig).

**Fig 3:**
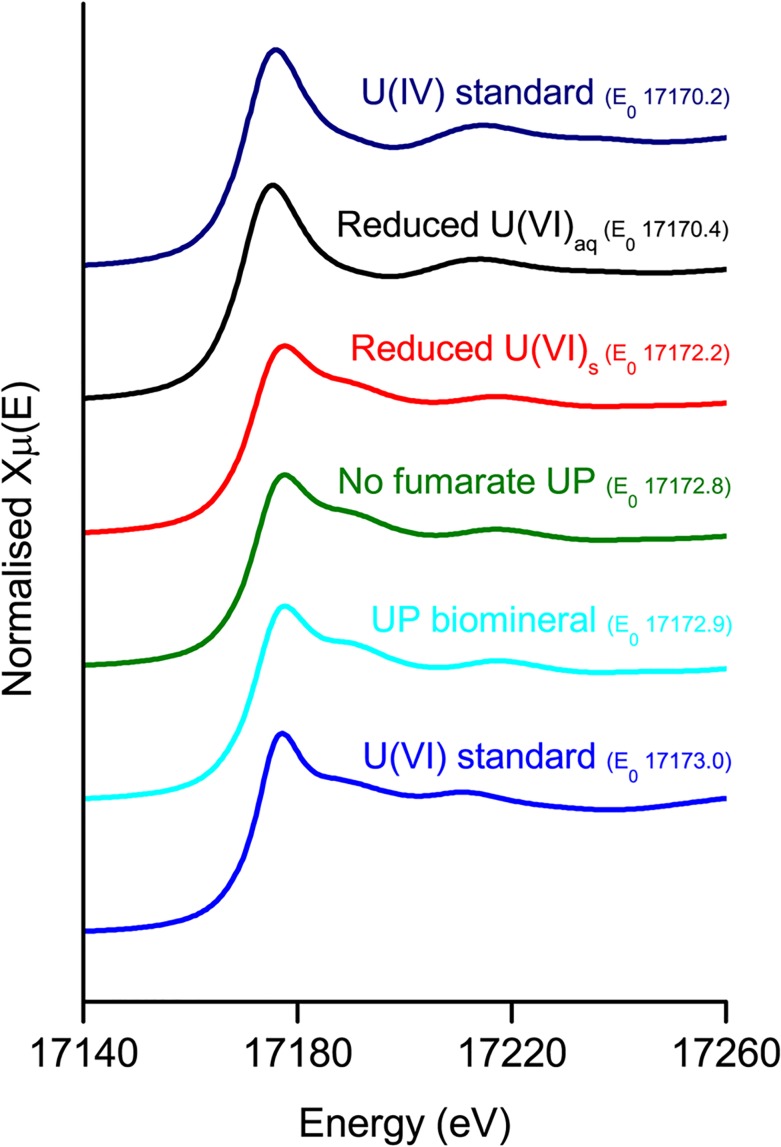
XANES spectra for *Serratia* biominerals, annotated with the edge position E_0_ (eV) obtained from the position of the first peak in the first derivative. Precipitates from the uranyl phosphate (UP) biomineralisation experiments display the same features as the U(VI) standard. The precipitate from the aqueous U(VI) reduction experiment is similar to the U(IV) standard while that from the solid U(VI) reduction experiment is intermediate.

The speciation and co-ordination of uranium in the minerals from the uranium-phosphate biomineralisation experiment and the “no fumarate” control were investigated by X-ray absorption spectroscopy. In this technique, the oxidation state of uranium can be determined by the position of the absorption edge and the shape of the XANES spectra. Information on the uranium co-ordination can be obtained from the EXAFS, by comparing the spectra with those for minerals with similar atomic configurations. For both samples, the XANES spectra displayed the characteristics features of U(VI) (Fig 3) suggesting they had been precipitated via a non-reductive mechanism. Given that autunite had been identified via XRD for both samples, the autunite crystal structure was used to fit the EXAFS spectra. Near-identical fits were obtained for the samples from the uranium-phosphate biomineralisation experiment and for the “no fumarate” control sample, suggesting that the precipitates from both experiments had the same uranium configuration. The fits comprised two axial and four equatorial oxygen atoms at 1.80 and 2.30 Å respectively and four monodentate phosphorus atoms at 3.63 or 3.66 Å, Both fits were improved significantly by including the contribution from eight U-O-P multiple scatterers at 3.70 Å. EXAFS fit parameters are provided in Table 1, and further discussion of EXAFS fitting is provided in the Supporting Information.

**Table 1:** Details of EXAFS fit parameters for *Serratia*-precipitated uranium biominerals; fitting details are proved in Supporting Information.

Sample	Path	Co-ordination number	Atomic distance (Å)	Debye-Waller factor σ^2^ (Å^2^)	Energy shift ∆E_0_ from calculated Fermi level (eV)	Reduced χ^2^	R “goodness of fit” factor	Confidence level of adding shell (α)^
**UP biomineral**	O _ax_	2	1.80 (1)	0.003 (1)	9.68 ± 1.69	4,600	0.038	-
	O _eq_	4	2.30 (1)	0.005 (1)				-
	P _monodentate_	4	3.66 (5)	0.014 (10)				0.91
	OP _ms_	8	3.69 (8)	0.018 (11)				0.76
**UP biomineral “no fumarate” control**	O _ax_	2	1.80 (1)	0.004 (1)	8.12 ± 1.28	12,900	0.030	-
	O _eq_	4	2.30 (1)	0.005 (1)				-
	P _monodentate_	4	3.63 (4)	0.011 (7)				0.93
	OP _ms_	8	3.70 (7)	0.017 (8)				0.73
**Microbial U(VI)**_**aq**_ **reduction**	O _eq1_	4	2.31 (2)	0.009 (2)	4.01 ± 0.71	110	0.017	-
	O _eq2_	4	2.44 (3)	0.015 (6)				0.91*
	P _bidentate_	2	3.13 (2)	0.015 (4)				0.98
	U	4	3.85 (1)	0.006 (1)				1.00
**Microbial U(VI)**_**s**_ **reduction**	O _ax1_	1.8	1.80 (1)	0.005 (1)	6.14 ± 1.93	1,750	0.031	-
	O _eq1_	4.4	2.28 (2)	0.007 (1)				-
	C	1.25	2.93 (4)	0.003 (5)				0.71
	P _monodentate_	3.5	3.65 (4)	0.012 (4)				0.92

Amplitude factor (S0^2^) was fixed at 1.0 for each sample. Numbers in parentheses are the SD on the last decimal place(s).

^ f-test results, α > 0.68 statistically improves fit with 1 sigma confidence, α > 0.95 with 2 sigma confidence

* This value is for splitting the shell of 8 equatorial oxygen into 4 and 4

These results clearly demonstrate the ability of this *Serratia* species to metabolise the glycerol phosphate donor under anaerobic conditions, releasing inorganic phosphate to solution and consequently precipitating uranyl phosphate minerals of the autunite group. Under these conditions this *Serratia* species was able to remove 1 mM of U(VI) within 25 days. This highlights the potential for biostimulation via glycerol phosphate addition to remediate uranium contamination in groundwater, under both anaerobic and aerobic conditions. The precipitation of uranyl phosphate in the “no fumarate” control suggests that the *Serratia* species may be able to ferment the glycerol phosphate, as the U(VI) added as the sole electron acceptor was not reduced. This demonstrates the potential flexibility of *Serratia* in a bioremediation scenario as it is able to precipitate U(VI) via multiple metabolic pathways.

### Microbial uranium(VI)_aq_ reduction

To determine whether the *Serratia* species was able to reduce U(VI) under growth conditions, cells were added to anaerobic freshwater minimal medium containing 1 mM U(VI), glycerol as the electron donor and following the results of the uranium solubility experiments, sodium trimetaphosphate as the source of phosphorus. A slight increase in optical density at 600 nm occurred after 5 days, but this was transient and was not present at day 8, nor throughout the remainder of the 43-day experiment (S4 Fig). U(VI) remained in solution and no precipitate was observed. Therefore, although the cells were able to grow in this medium in the absence of U(VI) (S1 Fig), they were not able reduce U(VI) under these conditions.

**Fig 4:**
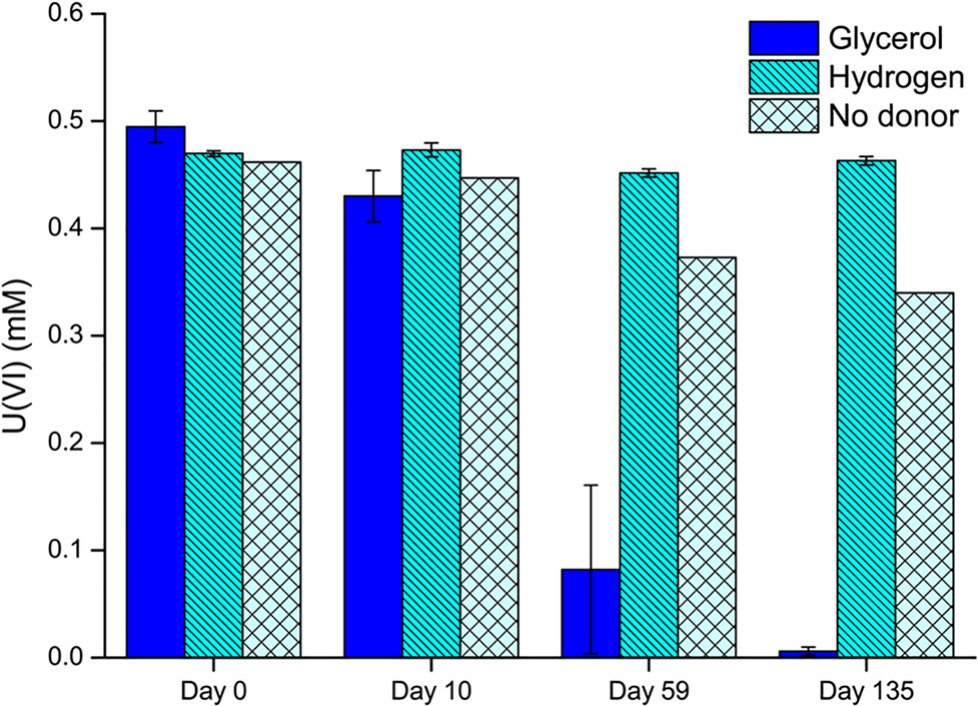
Removal of U(VI) from solution by resting cell suspensions of *Serratia*. Results obtained for cells with glycerol and AQDS and glycerol and fumarate were very similar to the results with glycerol so are not shown. Experiments were conducted in a bicarbonate buffer with glycerol or hydrogen as the electron donor and U(VI) as the electron acceptor. Each point represents the average of three replicates with error bars ± 1 standard deviation, except the no donor control which was a single measurement.

**Fig 5:**
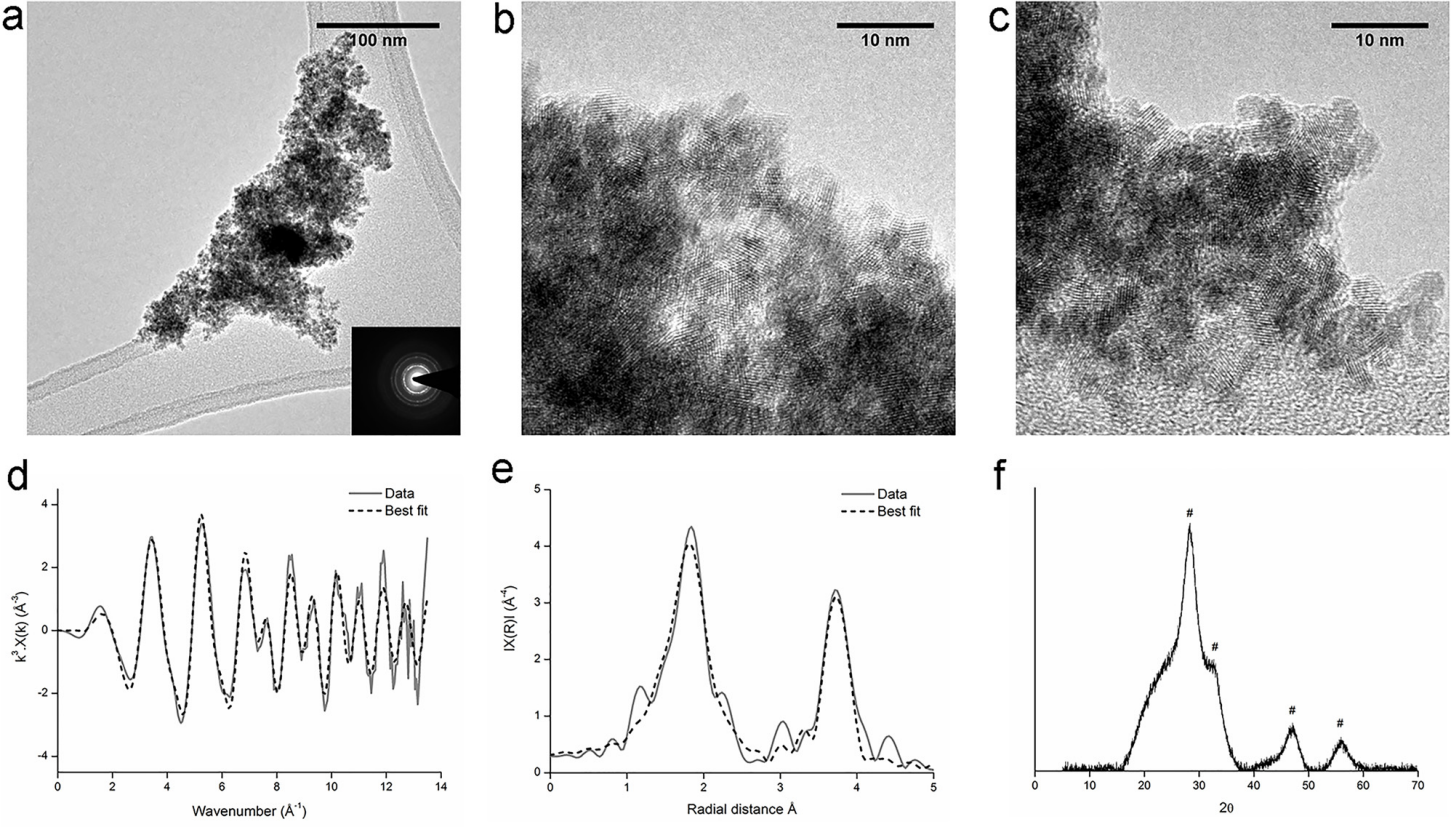
Microbial U(VI)_aq_ reduction experiment: TEM images (a, b, c), k^3^ weighted EXAFS data (d), non-phase shift corrected Fourier transform of EXAFS data (e), and XRD spectra (f). Dashed lines in XAS spectra represent the best fit of the data. # are peaks from uraninite (S6 Fig shows the peak pattern). Experiments were conducted in a bicarbonate buffer with glycerol as the electron donor and U(VI)_aq_ as the electron acceptor. Results confirmed the precipitate to be nanocrystalline uraninite.

Washed cell suspensions (resting cells) were used to further explore the reduction of U(VI). Cells were added to an anaerobic bicarbonate buffer at pH 7 to an optical density at 600 nm of 1.0, with glycerol or hydrogen as the electron donor and aqueous U(VI) as the electron acceptor. Almost all the U(VI) was removed from solution after 60 days in the incubations with glycerol (Fig 4) and grey/black precipitates were observed. By contrast the *Serratia* species appeared to be unable to use hydrogen as an electron donor for U(VI) reduction, perhaps suggesting that U(VI) reduction was linked to fermentation of glycerol. The grey/black precipitate from the experiments with glycerol as the electron donor was confirmed by XRD, TEM and SAED to be nanocrystalline uraninite (Fig 5).

Further characterisation using XAS revealed XANES spectra that contained the features characteristic of U(IV) (Fig 3). A good fit was obtained for the EXAFS spectrum using the crystal structure of uraninite with a small contribution from monomeric U(IV), a recently discovered form of non-crystalline U(IV) complexed to carboxyl or phosphoryl groups in biomass [32]. The fit included two shells of four equatorial oxygen atoms at 2.31 and 2.44 Å, two bidentate phosphorus atoms at 3.13 Å and four uranium atoms at 3.85 Å (Table 1, Fig 5). Note that while crystalline (bulk) uraninite has a U-U co-ordination of 12, nanoparticulate uraninite produces a peak with approximately 50% of the amplitude of the bulk phase, and can be fitted with a coordination number of 5.0 ± 1.9 [33], suggesting that the vast majority of U(IV) in thus sample was uraninite-like in character, with a small amount present as monomeric U(IV) complexed to phosphate-containing functional groups from biomass. Additional notes on EXAFS fitting are provided in the Supporting Information.

We believe this is the first time that a *Serratia* species has been shown to reduce U(VI), albeit under carefully controlled non-growth conditions. In comparison with the uranium phosphate biomineralisation experiments, the rates of uranium precipitation were slower, with 0.5 mM reduced over a 60 day period, although differences in experimental setup prevent direct comparison of their uranium removal efficiency. Nevertheless, these data highlight the metabolic diversity inherent within *Serratia* species and the roles that they could play in uranium bioremediation.

### Microbial uranium(VI)_s_ reduction

Following the successful demonstration of U(VI)_aq_ reduction by the *Serratia* species, experiments were carried out to assess whether the organism could reduce solid phase U(VI). Again washed “resting” cell suspensions were added to a bicarbonate buffer with glycerol as the electron donor, but with solid phase U(VI) as uranyl phosphate (from the uranium-phosphate biomineralisation experiments, Fig 2) as the sole electron acceptor. Following incubation for 70 days, visual inspection of the precipitates identified black “speckles” of material present within the bulk yellow uranyl phosphate (S5 Fig), and therefore the composition was further investigated using TEM, XRD and XAS.

**Fig 6:**
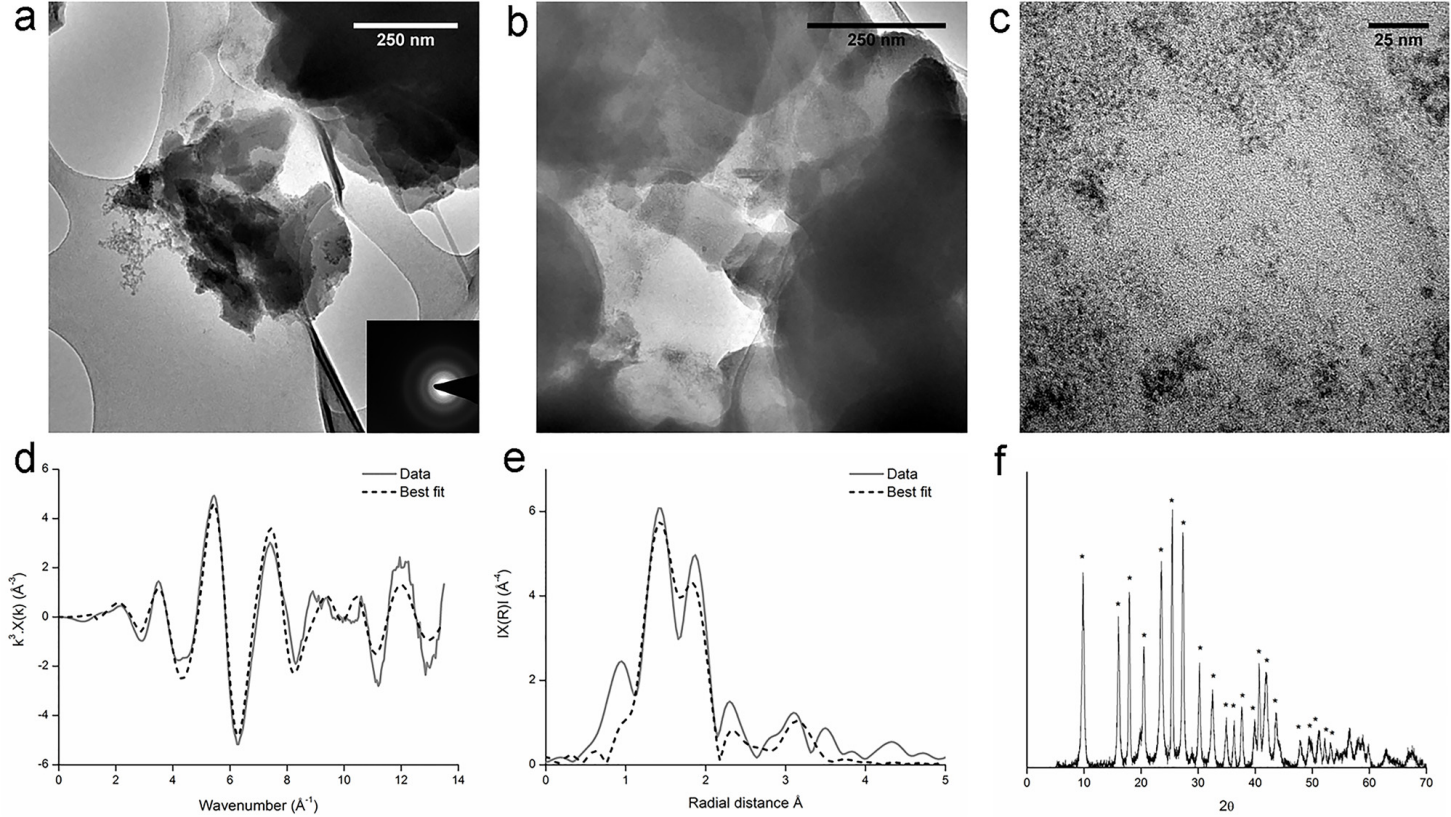
Microbial U(VI)_s_ reduction experiment: TEM images (a, b, c), k^3^ weighted EXAFS data (d), non-phase shift corrected Fourier transform of EXAFS data (e) and XRD spectra (f). Dashed lines in XAS spectra represent the best fit of the data. * are peaks from uranyl phosphates (S6 Fig shows the peak pattern). Experiments were conducted in a bicarbonate buffer with glycerol as the electron donor and microbially precipitated U(VI) phosphate (Fig 2) as the electron acceptor. Results confirmed the precipitate to be a uranyl phosphate biomineral of the autunite group, with some evidence for partial transformation to a uraninite-like U(IV) phase.

The majority of the TEM images were dominated by dense agglomerations of sheets of mineral (Fig 6), similar to the amorphous autunite group mineral that was added to these experiments as the starting material (Fig 2). However, a considerable number of areas comprised clusters of a different material, 2–3 nm in size (Fig 6) that resembled the uraninite from the U(VI) reduction experiments (Fig 5). This suggests that the original uranyl phosphate had been partially altered by the *Serratia* species. XRD analysis identified the mineral to be uranyl phosphate (Fig 6), although it is difficult for this technique to detect the presence of a small amount of nanocrystalline mineral within a bulk mineral phase.

Inspection of the XANES spectra for this sample showed a slightly lower edge position compared to the U(VI) standard (Fig 3) suggesting that a mixed phase may be present. Linear combination fitting was performed using the starting U(VI) material from the uranium-phosphate biomineralisation experiment and the U(IV) mineral from the microbial U(VI) reduction experiment as end members. Results revealed that 11% ± 0.9% had been reduced to U(IV) with the remaining uranium present as U(VI). However it should be noted that due to additional errors associated with linear combination fitting [34] this result is not conclusive, although it is supported by the observed shift in edge position and by the mineral alteration evident in the TEM images.

Given that the starting material was autunite; this model was initially used to fit the EXAFS spectra for samples from this experiment. Clearly the uranyl moiety contributed considerably to the first two shells, with characteristic peaks present at 1.78 and 2.28Å (Table 1, Fig 6). The co-ordination numbers were adjusted to take account of the results of the linear combination fitting which suggested that 10% of this sample was U(IV). The fit was statistically improved by the addition of a shell of P atoms at 3.65 Å, and also C at 2.93 Å representing a contribution from biomass. As the TEM images suggested that partial transformation to uraninite may have occurred, fits were attempted including U-U at 3.85 Å. However, no contribution from this shell was observed, nor the fit improved by its inclusion with any coordination number. Additional details of the EXAFS fitting procedure and statistical analysis are provided in the Supporting Information.

These data provide evidence that a *Serratia* species is able to reduce solid phase U(VI) to U(IV), although reduction was relatively inefficient. It is not possible to determine whether the solid-phase U(VI) was reduced directly, or whether it was first dissolved before being reduced (e.g. as discussed in Rui *et al*. 2013 [35]). The TEM images may suggest a dissolution mechanism, with the uraninite-like material present at distances of up to 100 nm from the dense sheets of the uranyl phosphate mineral, but this clearly warrants further attention.

## Conclusions

A *Serratia* species has been shown to be exhibit contrasting metabolic pathways capable of removing aqueous U(VI) from solution. This organism was able to respire or ferment glycerol phosphate consequently precipitating uranyl phosphate biominerals under anaerobic conditions. It could also reduce U(VI) to insoluble nanocrystalline uraninite under non-growth conditions using glycerol as the electron donor. Difficulties in experimental set up (i.e. growing versus resting cells) prevented direct comparison of the efficiency of the two processes, although the low solubility of uranyl phosphates over a wide range of environmental conditions means that uranyl phosphates are likely to be the better option for a long-term bioremediation strategy, given that uraninite can be subject to relatively facile oxidative remobilisation [36]. Some evidence for the transformation of solid U(VI) was observed, although it is perhaps more likely that limited dissolution occurred before microbial U(VI) reduction.

Future work could include testing the stability of *Serratia*-precipitated uranyl phosphate under conditions relevant to nuclear sites, perhaps by exposure to oxygenated groundwater, to see whether targeting phosphate biomineralisation does offer a long-term remediation solution. Additionally, as glycerol phosphate may be a cost-limiting factor to implementing uranium biomineralisation in the field [37,38] and given that sodium trimetaphosphate with glycerol was not a viable alternative, other organic phosphate sources could be investigated.

## Supporting Information

S1 Fig *Serratia* growth in anaerobic freshwater minimal medium. The freshwater minimal medium contained 4.3 mM PO_4_. The samples with trimetaphosphate contained an equivalent concentration. The samples with glycerol phosphate contained an additional 10 mM PO_4_. Each point represents the average of three replicates with error bars ± 1 standard deviation, except the no fumarate controls which were single measurements.
(EPS)

S2 Fig Monitoring of phosphate in the uranium-phosphate biomineralisation experiments. Experiments were conducted in an anaerobic freshwater minimal medium with glycerol phosphate as the electron donor and both fumarate and U(VI) as electron acceptors. The control contained no added fumarate. Phosphate release to solution indicated the rapid use of glycerol phosphate when fumarate was included as an electron acceptor. Each point represents the average of three replicates with error bars ± 1 standard deviation, except the no fumarate control which was a single measurement.
(EPS)

S3 Fig Uranium-phosphate biomineralisation “no fumarate” control experiment: TEM images (a, b, c), k^3^ weighted EXAFS data (d), non-phase shift corrected Fourier transform of EXAFS data (e), and XRD spectra (f). Experiments were conducted in an anaerobic freshwater minimal medium with glycerol phosphate as the electron donor and U(VI) as the electron acceptor. Dashed lines in XAS spectra represent the best fit of the data. * are peaks from uranyl phosphate (S6 Fig shows the peak pattern).
(EPS)

S4 Fig *Serratia* growth in anaerobic freshwater minimal medium with trimetaphosphate. Glycerol was included as the electron donor and U(VI) and fumarate as the electron acceptors. Each point represents the average of three replicates with error bars ± 1 standard deviation. The data are presented at the same scale as Fig 1.
(EPS)

S5 Fig Photographs of the microbial U(VI)_s_ reduction experiment after 70 days incubation. Experiments were conducted in a bicarbonate buffer with glycerol as the electron donor and microbially precipitated U(VI) phosphate (Fig 2) as the electron acceptor. Black “speckles” were observed within the bulk yellow uranyl phosphate mineral; some have been highlighted in the red circles in the enlarged imaged.
(EPS)

S6 Fig XRD spectra with peak patterns for uranyl phosphates or uraninite. Peak patterns for uranyl phosphate minerals are illustrated for the uranium-phosphate biomineralisation, the “no fumarate” control and the microbial U(VI)_s_ reduction precipitates. The peak pattern for uraninite is illustrated for the microbial U(VI)_aq_ reduction precipitate.
(EPS)

S1 File Supporting Information–Details of EXAFS Fitting. (DOCX)
