# Supplementary material for: Uranium Biominerals Precipitated by an Environmental Isolate of Serratia under Anaerobic Conditions
Source: PLoS One. 2015 Jul 1;10(7):e0132392. doi: 10.1371/journal.pone.0132392 (PMC4488441; doi:10.1371/journal.pone.0132392)
Supplement: S1 File — (DOCX) [file pone.0132392.s007.docx]

# Supporting Information – Details of EXAFS Fitting

EXAFS fitting was performed using ARTEMIS, with published crystal structures for autunite, nanocrystalline uraninite or monomeric U(IV). The effect of varying coordination numbers was assessed during the fitting process; values were selected based on those which obtained the best fitting parameters. Additional shells were only included if they statistically improved the fit.

## Uranium-phosphate biomineralisation

The crystal structure of autunite was used to as a model for fitting the spectra for the uranium-phosphate biomineralisation precipitates. For both minerals the first two peaks fitted well with 2 axial and 4 equatorial oxygen atoms at 1.80 Å and 2.30 Å, as expected from U(VI) uranyl. Inclusion of a shell of 4 phosphorus atoms at 3.63 Å (uranium-phosphate biomineralisation) or 3.66 Å (uranium-phosphate biomineralisation no fumarate control) statistically improved the fits with 1 sigma confidence and consistent with an autunite like coordination environment. Oxygen-oxygen multiple scattering statistically improved the fit but lead to unacceptably large changes in atomic distances from those in the autunite crystal structure and thus was excluded. By contrast, oxygen-phosphorus multiple scattering significantly improved the fits (1 sigma confidence). Data for the best fits are presented in Fig. 2 and S3 Fig***.*** and the fitting parameters are listed in Table 1 and S1 Table.

Attempts were made to add additional shells of single scatterers including O at 3.89 Å and Na at 4.12 Å from the autunite crystal structure, and also C at 2.91 Å to represent a contribution from biomass. While these improved the fitting parameters (reduced χ^2^ and R-factor), their inclusion could not be justified due to relatively high errors on the Debye-Waller factors. This is perhaps unsurprising due to these light elements being relatively poor scatterers, and present at some distance from the central U atom. However, visible inspection of the spectra shows peaks present at 2.83 Å and 4.05 Å which are not well represented by the fit; it is likely that C and O/Na contributed to these peaks despite it not being possible to include them in the fit due to physical or statistical limitations.

## Microbial U(VI)_aq_ reduction

The crystal structure of nano uraninite was used to fit the spectra for the precipitate from the microbial U(VI)_aq_ reduction experiment. The first peak was initially fitted with eight oxygen atoms. Addition of four U at 3.85 Å statistically improved the fit with 3 sigma confidence. This fit was further improved by including a contribution from biomass, with the best fit for 2 P atoms at 3.13 Å. This suggests that the sample is mostly nanocrystalline uraninite, but also contains a small fraction of “monomeric” U(IV). Finally, splitting the oxygen shell to two shells of four O at 2.31 and four at 2.44 Å statistically improved the fit with 1 sigma confidence. This type of fit is not without precedent; a number of published EXAFS spectra for biologically-precipitated U(IV) have also been fitted with a split oxygen shell, such as in Alessi et al. 2012 and Boyanov et al. 2011. The best fit is presented in Fig. 5 and the fitting parameters are listed in Table 1 and S1 Table.

## Microbial U(VI)_s_ reduction

Given that the starting material was the uranyl phosphate from the uranium-phosphate biomineralisation experiment, the crystal structure of autunite was used to fit the spectra from the microbial U(VI)_s_ reduction experiment. The co-ordination of the oxygen shells was adjusted to reflect results of linear combination fitting that suggested the sample contained around 10 % U(IV). A precipitate containing 10 % U(IV) and 90 % U(VI) would have 1.8 axial oxygen atoms, 4 equatorial oxygen atoms at 2.30 Å and 0.4 equatorial atoms at 2.44 Å. Changing the co-ordination from 2 axial and 4 equatorial oxygen atoms to 1.8 axial and 4.4 equatorial improved the fitting parameters, but addition of an extra shell at 2.44 Å could not be justified. The fit was improved by adding 4 phosphorus atoms at 3.70 Å consistent with the autunite crystal structure.

Again attempts were made to add additional shells of single scatterers including O at 3.89 Å and Na at 4.12 Å from the autunite crystal structure, and also C at 2.91 Å to represent a contribution from biomass. In this case the fit was improved by adding 1.25 bidentate carbon atoms at 2.93 Å and reducing the phosphorus co-ordination to 3.5. Although the Debye-Waller factor for carbon was relatively low (0.003) and consequentially the error relatively high (0.005), this shell was included due to the statistical improvement of the fit and its plausible nature due to the high concentrations of biomass in the experiments. The further addition of O, Na, multiple scattering or U shells could not be justified due to physical or statistical limitations. The best fit which represents a U(VI) phosphate dominated system with a small component of U(IV) is presented in Fig. 6 and fitting parameters are provided in Table 1 and S1 Table.

## Supporting tables

## S1 Table – Additional EXAFS fitting parameters

| **Sample** | **Number of independent variables** | **Number of variables used in the fit** | **Degrees of freedom** | **k-range** | **R-range** |
| --- | --- | --- | --- | --- | --- |
| UP biomineral | 17.8 | 8 | 9.8 | 3.0 – 13.5 | 1.0 – 3.7 |
| UP biomineral “no fumarate” control | 17.8 | 8 | 9.8 | 3.0 – 13.5 | 1.0 – 3.7 |
| Microbial U(VI)_aq_ reduction | 19.4 | 9 | 10.4 | 3.0 – 13.5 | 1.3 – 4.25 |
| Microbial U(VI)_s_ reduction | 16.7 | 9 | 7.7 | 3.0 – 13.5 | 1.15 – 3.7 |

All fits were performed in R space

## Supporting references

Alessi DS, Uster B, Veeramani H, Suvorova EI, Lezama-Pacheco JS, Stubbs JE, et al. Quantitative separation of monomeric U(IV) from UO_2_ in products of U(VI) reduction. Environ Sci Technol. 2012;46: 6150–6157. doi:10.1021/es204123z

Boyanov MI, Fletcher KE, Kwon MJ, Rui X, O’Loughlin EJ, Loffler FE, et al. Solution and microbial controls on the formation of reduced U(IV) species. Environ Sci Technol. 2011;45: 8336–8344. doi:10.1021/es2014049
